# Crystal structure of the atypically adhesive SpaB basal pilus subunit: Mechanistic insights about its incorporation in lactobacillar SpaCBA pili

**DOI:** 10.1016/j.crstbi.2020.11.001

**Published:** 2020-12-08

**Authors:** Abhin Kumar Megta, Shivendra Pratap, Abhiruchi Kant, Airi Palva, Ingemar von Ossowski, Vengadesan Krishnan

**Affiliations:** a Laboratory of Structural Microbiology, Regional Centre for Biotechnology, NCR Biotech Science Cluster, Faridabad, 121001, India; b School of Biotechnology, KIIT University, Odisha, 751024, India; c Department of Biotechnology, Manipal University, Karnataka, 576104, India; d Department of Veterinary Biosciences, University of Helsinki, Helsinki, FIN-00014, Finland

**Keywords:** Sortase-dependent SpaCBA pili, SpaB basal pilin, Cell-wall anchoring, Mucus adhesion, *Lactobacillus rhamnosus* GG, Probiotic, Ig, immunoglobulin, Cna, collagen adhesin, ECM, extracellular matrix, MD, molecular dynamics, PDB, Protein Data Bank, MS, mass spectrometry, rmsd, root mean square deviation, ABC, ammonium bicarbonate, ACN, acetonitrile, PEG, polyethylene glycol

## Abstract

To successfully colonize a host or environment, certain genera and species of Gram-positive bacteria have evolved to utilize the so-called sortase-dependent pilus, a long multi-subunit and non-flagellar surface adhesin. One example of this is *Lactobacillus rhamnosus* GG, a gut-adapted probiotic strain that produces SpaCBA pili. These structures are covalent hetero-oligomers built from three types of pilin subunit, each with a specific location and function (i.e., backbone SpaA for length, tip SpaC for adhesion, and basal SpaB for anchoring). Functionally, the SpaCBA pilus exhibits a promiscuous affinity for components on intestinal surfaces (e.g., mucus, collagen, and epithelial cells), which is largely attributed to the SpaC subunit. Then again, the basal SpaB pilin, in addition to acting as the terminal subunit during pilus assembly, displays an out of character mucoadhesive function. To address the structural basis of this unusual dual functionality, we reveal the 2.39 ​Å resolution crystal structure of SpaB. SpaB consists of one immunoglobulin-like CnaB domain and contains a putative intermolecular isopeptide bond-linking lysine and internal isopeptide bond-asparagine in an FPKN pilin motif within the C-terminal end. Remarkably, we found that a C-terminal stretch of positively charged lysine and arginine residues likely accounts for the atypical mucoadhesiveness of SpaB. Although harboring an autocatalytic triad of residues for a potential internal isopeptide interaction, the SpaB crystal structure lacked the visible electron density for intact bond formation, yet its presence was subsequently confirmed by mass spectral analysis. Finally, we propose a structural model that captures the exclusive basal positioning of SpaB in the SpaCBA pilus.

## Highlights

• The crystal structure of the *L. rhamnosus* GG SpaB basal pilin has been determined at 2.39 ​Å resolution.
• SpaB is comprised of a single immunoglobulin-like CnaB, which harbors a spontaneously formed internal isopeptide bond and a putative linking lysine in a conventional FPKN pilin motif.
• The structural basis for the atypical mucoadhesiveness of SpaB is explained.
• A molecular model of the entire SpaCBA pilus structure is constructed and shows the exclusive basal positioning of SpaB.

## Graphical abstract

**Figure:**
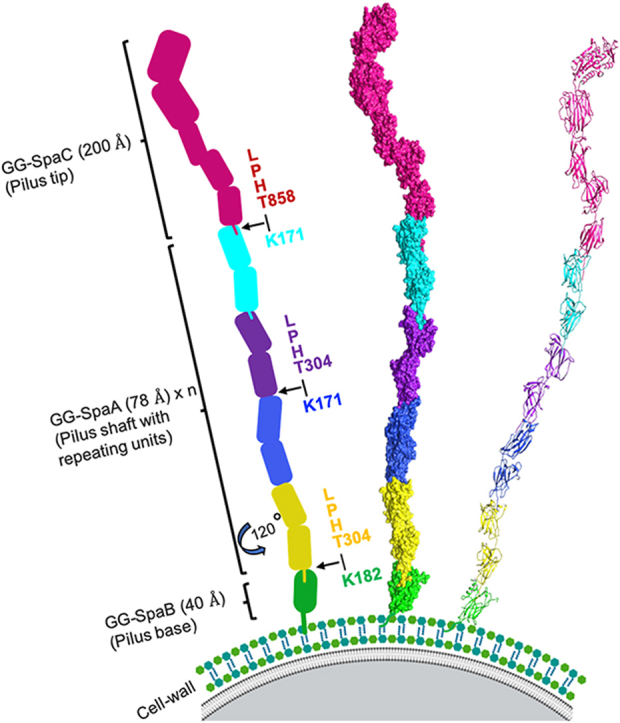

## 1 Introduction

To survive within highly competitive microbial ecosystems, bacteria have evolved a variety of different mechanisms to promote their targeted adhesion and subsequent colonization of host environments. Regarding Gram-positives, certain genera and species exhibit so-called ‘sortase-dependent’ pili (*sing*., pilus), which characteristically appear as long and adhesive non-flagellar proteinaceous protrusions jutting out from the bacterial cell surface (Khare and Narayana, 2017; Pansegrau and Bagnoli, 2017; Telford et al., 2006; Ton-That and Schneewind, 2004; von Ossowski, 2017). By virtue of this extended nature, bacteria with a sortase-dependent pilus can make first contact with the immediate surroundings, giving them a distinct edge over any non-piliated competitors and an effective factor for virulence activity (pathogens) (Danne and Dramsi, 2012; Mandlik et al., 2008b; Telford et al., 2006) or niche adaptation (commensals) (Kankainen et al., 2009; Kant et al., 2014; Krishnan et al., 2016; Lebeer et al., 2009; Turroni et al., 2013; Yu et al., 2015).

The archetypal structural organization of these sortase-dependent pili is as hetero-oligomeric covalent polymers (Ramirez et al., 2020), in which two or three (usually three) types of pilin subunit are connected linearly head-to-tail, each with its own defined location and function, i.e., backbone pilin for length, tip pilin for adhesion, and basal pilin for anchoring (Hilleringmann et al., 2009). True to the name, the pilin-specific C-type sortase enzyme catalyzes a succession of pilins into a polymeric structure through the formation of a covalent intermolecular isopeptide bond between two key motif residues within adjacent subunits (Hendrickx et al., 2011; Mandlik et al., 2008b; Siegel et al., 2016). This involves the side chain ε-amino group of the ‘linking’ lysine (K) in the YPKN pilin motif of the N-terminal region (head) in one pilin and the carbonyl-group carbon of the threonine (T) in the LPXTG motif of the C-terminal sorting-signal region (tail) in another pilin. Expectedly, the backbone pilin, which has both peptide motifs, represents most subunits in the growing pilus structure. The adhesive tip pilin, which typically lacks the YPKN pilin motif, is only positioned at the beginning of the pilus (Hilleringmann et al., 2009). The basal pilin is primarily deposited at the pilus base, though in some instances it possesses both motifs and can also be found along the pilus backbone (Mandlik et al., 2008b). Pilus elongation normally comes to an end with the appearance of the basal pilin being carried by the housekeeping A-type sortase. Here, the basal subunit is subsequently connected to the last backbone pilin of the pilus structure via a C-type sortase-catalyzed K-T isopeptide bond (Necchi et al., 2011; Swaminathan et al., 2007). As a final step to anchoring the fully assembled pilus, the A-type sortase catalyzes a covalent link between the LPXTG-threonine of the basal subunit and the peptidoglycan layer of the cell wall (Chang et al., 2019; Mandlik et al., 2008a). Here, the incorporation of the basal pilin is thought to act as the possible signal that ends pilus polymerization (Mandlik et al., 2008a). Finally, to ensure that pilus protein production is in step with the overall polymerization process, the genes for the backbone, tip, and basal pilins and the C-type sortase are always operonic in the bacterial genome (Mandlik et al., 2008b). The A-type sortase gene is the exception and occurs elsewhere along the genome.

X-ray crystallography has revealed that the tip, basal, and backbone subunits are modular in structure and mainly consist of CnaA and CnaB domains (Kang and Baker, 2012; Krishnan, 2015), both of which are variant immunoglobulin (Ig)-like folds of the staphylococcal collagen adhesin (Cna) (Deivanayagam et al., 2000; Symersky et al., 1997). Whilst the core fold of the CnaA and CnaB domains is conserved, comprising nine and seven β-strands, respectively (Vengadesan and Narayana, 2011), there are some additional variations in the topologies that distinguish each pilin. Tip pilins are the largest, consisting of N-terminal binding and C-terminal stalk-like regions that include a globular domain and three or four CnaA/CnaB domains (Izore et al., 2010; Kant et al., 2020; Krishnan et al., 2013; Linke-Winnebeck et al., 2014; Pointon et al., 2010). Backbone pilins have a two-to-four domain structure that includes a mix of the CnaA and CnaB folds (Krishnan, 2015). The N-terminal domain, which contains the linking lysine, is highly flexible in nature in order to facilitate the head-to-tail joining of adjacent backbone subunits during pilus assembly (Vengadesan and Narayana, 2011). As the smallest pilin, the basal subunits have a structure that includes one to three CnaB domains (Krishnan, 2015). Uniquely to the majority of basal pilins, a segment of the C-terminal tail region of these subunits is rich in hydrophobic proline residues (Krishnan et al., 2007; Linke et al., 2010; Shaik et al., 2014) and might have an involvement in the anchoring of the pilus onto the cell wall (Linke et al., 2010). For all three pilin types, an internal isopeptide bond in the CnaA and CnaB domains provides an element of increased and strengthened rigidity in the folded structure (Kang and Baker, 2009; Kang et al., 2007). However, unlike the intermolecular K-T isopeptide interaction (i.e., a sortase-catalyzed transpeptidation), these internal (intra-domain) isopeptide bonds occur spontaneously and require the triad configuration of the lysine (K), asparagine/aspartate (N/D), and autocatalytic glutamate (or aspartate) residues in a hydrophobic environment. Here, an autocatalytic K–N (or K-D) isopeptide bond forms when the non-protonated side chain ε-amino group of lysine initiates a nucleophilic attack on the side chain carbon (Cγ) of asparagine (or aspartate), and for which the nearby acidic glutamate (or aspartate) serves as the proton shuttle.

For some time, we have delved into solving the crystal structures of the pilin (Chaurasia et al., 2016, 2018; Kant et al., 2016, 2020; Kumar Megta et al., 2019; Megta et al., 2019; Mishra et al., 2017; Singh et al., 2013) and sortase (Pratap et al., 2019) proteins from *Lactobacillus rhamnosus* GG, a strongly adapted gut-transient probiotic strain and one of just a few known commensal bacteria with sortase-dependent pili (Kankainen et al., 2009; Kant et al., 2014; Lebeer et al., 2009; Turroni et al., 2013; Yu et al., 2015). *L. rhamnosus* GG contains the *spaCBA* operon (*spaC*-*spaB*-*spaA*-*srtC1*) that produces the so-called SpaCBA pilus, which is comprised of the tip SpaC, basal SpaB, and backbone SpaA pilins (Kankainen et al., 2009; Reunanen et al., 2012). A second operon, called *spaFED* (*spaF*-*spaE*-*spaD*-*srtC2*), for another type of pilus (SpaFED) is also present in *L. rhamnosus* GG, but its expression has only been established recombinantly in *Lactococcus lactis* (Rintahaka et al., 2014), and thus whether a native form is producible in this strain or others remains unconfirmed (Reunanen et al., 2012). Research into the molecular mechanisms that underlie the intestinal adaptation and probiosis of *L. rhamnosus* GG has revealed the SpaCBA pilus (and potentially the SpaFED pilus) is one of the ways by which its transient gut colonization can be prolonged (von Ossowski, 2017). This is largely attributed to the adhesive nature of the SpaCBA pilus (via its SpaC tip pilin) to, e.g., intestinal mucus (Kankainen et al., 2009; von Ossowski et al., 2010; von Ossowski et al., 2013), collagen (Tripathi et al., 2013), and intestinal epithelial cells (Ardita et al., 2014; Lebeer et al., 2012). In a further continuation of our attempts to understand the mechanistic processes behind the assembly of the sortase-dependent SpaCBA pilus, we now focus on the structural determination of the basal SpaB subunit from *L. rhamnosus* GG. While SpaB displays the representative attributes of a basal pilin, its recombinant form demonstrates an atypical adhesiveness for intestinal mucus that is sevenfold greater than observed with either SpaC or SpaF (von Ossowski et al., 2010). Conversely, the basal SpaE subunit lacks the same binding ability toward mucus glycans. Since SpaB shares no homology with any known mucus-binding proteins, the structural basis for mucoadhesiveness might lie with its alkaline isoelectric point, which, as a positively charged protein, would allow for electrostatic interactions to occur between negatively charged mucus (von Ossowski et al., 2010). By comparison, the other SpaCBA and SpaFED pilin subunits are acidic proteins that would not interact similarly, though SpaC and SpaF are both mucus binders (von Ossowski et al., 2010). However, since SpaB makes no added contribution to the binding ability of the SpaCBA pilus (von Ossowski et al., 2010; von Ossowski et al., 2013), the biological relevance of its mucoadhesiveness remains unknown.

We now report the crystal structure of the basal SpaB pilin at 2.39 ​Å resolution. SpaB consists of a single CnaB domain with a putative linking lysine in a conserved FPKN pilin motif at the C-terminal end of the protein. While SpaB harbors the residues for a potential internal K–N isopeptide interaction, there was insufficient electron density to support the formation of an intact bond, though we provide evidence that it occurs in solution. Noticeably, the C-terminal tail region of SpaB contains a markedly lower number of prolines than those of other basal pilins, but, on the other hand, it is enriched with lysine and arginine residues that likely impart a net positive charge to the protein surface that facilitates binding to intestinal mucin. Lastly, we propose a structural model that brings new molecular insights into how the basal SpaB subunit is incorporated into the SpaCBA pilus.

## 2 Materials and methods

### 2.1 Protein production and crystallization

Full-length *L. rhamnosus* GG SpaB (GG-SpaB_FL_) protein (residues 33–205) was solubly produced in *Escherichia coli* (von Ossowski et al., 2010), essentially as done previously for the GG-SpaA (Singh et al., 2013), GG-SpaC (Kant et al., 2016), GG-SpaD (Chaurasia et al., 2015), and GG-SpaE (Mishra et al., 2017) pilins. Since the GG-SpaB_FL_ protein (~20 ​kDa) did not yield diffraction quality crystals, a truncated version (GG-SpaB_C-trun_) missing a flexible portion of the C-terminal region (residues 185–205), but containing N-terminal hexahistidine tagging was cloned and solubly produced in *E. coli* as described previously (Kumar Megta et al., 2019). Homogeneous and pure GG-SpaB_C-trun_ protein (residues 31–184) yielded hexagonal crystals after the surface lysine methylation (SLM) treatment and the inclusion of 0.2 ​M MgCl_2_ as an additive (Kumar Megta et al., 2019). The SLM treatment was done as described previously (Kumar Megta et al., 2019; Rayment, 1997). Briefly, GG-SpaB protein in HEPES buffer (50 ​mM HEPES pH 7.5, 250 ​mM NaCl) was concentrated to a final volume of 10 ​ml (~1 ​mg/ml) and then supplemented with aliquots of freshly prepared 1 ​M borane-dimethylamine complex (200 ​μl) (Sigma-Aldrich) and 36.5–38% formaldehyde solution (400 ​μl) (Sigma-Aldrich), followed by gentle shaking (100 ​rpm) at 4 ​°C for 2 ​h. This step was repeated. A final 100 ​μl aliquot of 1 ​M borane-dimethylamine complex was added to the mixture and allowed to incubate overnight at 4 ​°C with gentle shaking. The mixture was centrifuged to remove any precipitant and then concentrated to a final ~900 ​μl volume for size-exclusion chromatography using a HiPrep 26/60 Sephacryl S-200 HR column (GE healthcare) equilibrated in Tris buffer (20 ​mM Tris-Cl pH 8.0, 150 ​mM NaCl, 1 ​mM DTT). Eluted fractions of GG-SpaB protein were pooled and then concentrated to 40 ​mg/ml for crystallization trials. Here, crystals for X-ray diffraction experiments were obtained from lysine-methylated GG-SpaB_C-trun_ protein (40 ​mg/ml in 20 ​mM Tris–HCl pH 8.0, 150 ​mM NaCl, 1 ​mM DTT) using a screening solution of 0.2 ​M MgCl_2_, 0.1 ​M MES pH 6.5, 30% (w/v) PEG 4000 (Kumar Megta et al., 2019).

### 2.2 X-ray data collection, processing, and structure determination

Native high-resolution X-ray diffraction data were obtained from lysine-methylated GG-SpaB_C-trun_ crystals using a 30% PEG 600 cryoprotectant. An anomalous dataset for single-wavelength anomalous dispersion (SAD) phasing was also obtained from an iodide-derivative crystal (soaked for 3 ​min in a cryosolution of 30% (v/v) ethylene glycol and 500 ​mM sodium iodide) (Kumar Megta et al., 2019). Native and anomalous datasets (wavelength 0.97872 and 1.7712 ​Å, respectively) were collected at a synchrotron on beamline BM14 (ESRF, Grenoble, France). Diffraction data were indexed and integrated with *XDS* (Kabsch, 2010) and scaled with *AIMLESS* (Evans and Murshudov, 2013) using the autoPROC package (Vonrhein et al., 2011). During initial data processing, POINTLESS scores were indicative of a 622 point-group symmetry. An attempt to resolve the ambiguity in space-group assignment was done by merging and scaling the data in all possible point groups (*P*6_1_22, *P*6_2_22, *P*6_3_22, and *P*6_5_22) and then determining the structure via molecular replacement (MR) and/or iodide-SAD. These initial efforts failed likely due to the low sequence identity (<28%) between GG-SpaB and known structures or a weak anomalous signal. Later, a total of 25 ​MR search models were generated by creating a custom library of either four- or three-stranded β-sheet core regions derived from distant structural homologs with a CnaB domain. A MR attempt yielded structure solution in space group *P*6_5_22. After building all residues but excluding those for the AB loop, which was disordered, the R_work_/R_free_ was 0.28/0.32. Next, the possibility of lower symmetry space group was examined by processing the data in space group *P*3, which, according to a Matthew's coefficient analysis, indicated eight molecules in the asymmetric unit with 43% solvent content (*V*_M_ ​= ​2.17 ​Å^3^ Da^−1^, *V*_S_ ​= ​43%). X-ray structure solution via the MR technique was attempted with all possible space groups using the initial model obtained from the data with *P*6_5_22. The best structure solution was obtained in the *P*3_2_ space group with PHASER (McCoy et al., 2007) in *PHENIX* (Adams et al., 2010). Final refinement of this model gave R_work_/R_free_ values of 0.19/0.23. The final model was assessed for structure quality and validation via COOT (Emsley et al., 2010) and PBD tools. Coordinates for the refined structure have been deposited in the PDB under the ID code 7CBS.

### 2.3 Mass spectrometry analysis

Mass spectrometry analysis for the presence of an internal K–N isopeptide bond in recombinant GG-SpaB_FL_ and GG-SpaB_C-trun_ proteins was done using the electrospray ionization (ESI) triple TOF 5600 mass spectrometer (SCIEX). Protein purity prior to mass spectrometry was judged by SDS-PAGE (15%) (Fig. S1). Here, the protein bands were excised from an SDS-polyacrylamide gel and exposed to repetitive cycles of dehydration and rehydration with 50% acetonitrile (ACN) and 25 ​mM ammonium bicarbonate (ABC) to remove protein staining. Excised gel slices suspended in 25 ​mM ABC were then incubated at 37 ​°C with 500 ​ng trypsin (Promega) for 4 ​h and 200 ​ng AspN endopeptidase (Roche) for 16 ​h. Digested peptides were run on a C18 column equilibrated with 100% ACN followed by 0.1% aqueous formic acid and then afterward eluted by a linear 35–70% gradient of 0.1% aqueous formic acid in ACN. Mass spectrometric data of the peptides were acquired within the m/z range using Analyst® software (SCIEX) and interpreted using PeakView® 2.2 software (SCIEX). During the peak assignment to identify cross-linked peptides representing isopeptide bonds, the Bio Tool Kit within PeakView® 2.2 software and the MS/MS Fragment Ion Calculator (http://db.systemsbiology.net:8080/proteomicsToolkit/FragIonServlet.html) were used for calculating the mass of peptide fragment ions. Initially, mass spectrometry analysis of the excised bands corresponding to freshly purified samples of GG-SpaB_FL_ and GG-SpaB_C-trun_ gave no indication of isopeptide bond formation (Fig. S1). On the other hand, GG-SpaB_FL_ protein that had been stored at 4 ​°C for two days appeared to migrate on SDS-polyacrylamide gels as a doublet band, a phenomenon previously attributed to internal isopeptide bond formation in other recombinant pilin proteins such as GG-SpaA (Chaurasia et al., 2016). Thus, in-solution digestion of the doublet band using the same protocol as used for in-gel digestion (see above) but excluding the dehydration/rehydration cycle, followed by mass spectrometry analysis was subsequently undertaken. Here, a cross-linked peptide containing an internal isopeptide bond was detected in the stored sample of GG-SpaB_FL_ protein.

### 2.4 Biolayer interferometry binding assay

Biolayer interferometry was used to estimate the binding affinity between GG-SpaB (GG-SpaB_FL_ and GG-SpaB_C-trun_) and porcine gastric mucin (Type II) (Sigma-Aldrich) using a ForteBio Octet Red 96 instrument (ForteBio, Inc) equipped with aminopropylsilane (APS) biosensors. Mucin type II stock in PBS buffer (10 ​mM sodium phosphate pH 7.4, 137 ​mM NaCl, 2.7 ​mM KCl) was brought up to a 4 ​mg/ml concentration. Initial optimizations for the binding assay were done to determine the ligand (GG-SpaB) and analyte (mucin) concentrations and the experimental temperature and pH conditions. Here, experiments were carried out in PBS buffer at 30 ​°C. APS biosensor tips were hydrated in PBS buffer for 600 ​s, immobilized with 10 μM GG-SpaB protein, and washed with PBS buffer. As a control, 1.5 ​μM bovine serum albumin (BSA) was also immobilized to omit the binding of analyte (mucin) during the association step. After a washing step with PBS buffer, a 200 ​μl aliquot of mucin Type II (~19.5–312 ​nM) was added to a 96-well micro plate (Greiner Bio-One, Germany), which was then mixed via 1000 ​rpm rotation and afterward allowed to interact with the immobilized GG-SpaB (~600 ​s association and ~900 ​s dissociation). Biosensors were repeatedly neutralized with PBS buffer and regenerated with 10% sodium dodecyl sulfate (SDS) for additional measurements. All binding experiments were performed in duplicate. Data acquisition and interpretation were performed with ForteBio data analysis 10.0 software.

### 2.5 Molecular simulations and protein-protein docking

Molecular dynamics simulation of the GG-SpaB models was performed with the AMBER14 program package (http://ambermd.org/). AMBER14 force field and TIP3P (Transferable Intermolecular Potential with 3 Points) were used for analyzing proteins and water molecules, respectively. Model of the GG-SpaB crystal structure was hydrated in a 10 ​Å cubic water box, along with the addition of 11 Na^+^ ions for net charge neutralization. Heating to 300 ​K was done incrementally, and the simulation runs were for 100 ns and coupled with a ramp-up time of 10 fs. Temperature and pressure were controlled by using a Nosé-Hoover thermostat (coupling constant *t*_t_ ​= ​2.524, 25) and a Parrinello-Rahman barostat (*t*_p_ ​= ​5.0 ps), respectively. Measurements of AB loop flexibility (Cα atoms and amino-acid residues) by root mean-square fluctuations (RMSF) and B-factor calculations per simulation frame were analyzed by using CPPTRAJ (http://ambermd.org/tutorials/analysis/#cpptraj) with visualization via VMD (http://www.ks.uiuc.edu/Research/vmd/). Graphical representation of the RMSF and B-factor values were plotted with GraphPad Prism 8 software (GraphPad Software Inc., San. Diego, USA).

Computational protein-protein docking experiments were carried out with the PIPER (Kozakov et al., 2006) interface of the BioLuminate tool from the Schrödinger software suite (https://www.schrodinger.com/). Crystal structures of the C-terminal domain of backbone GG-SpaA (GG-SpaA_Cdom_; PDB ID: 5F44) and basal GG-SpaB (this study) posed as the ligand (tail) and receptor (head), respectively. The first top model among the poses was selected. To generate the GG-SpaA/GG-SpaB complex, the full-length structure of GG-SpaA (PDB ID: 5F44) was superposed onto that of GG-SpaA_Cdom_ via COOT (Emsley et al., 2010).

### 2.6 In silico characterizations

Protein disorder predictions using primary structures were run on the DISOPRED2 Disorder Prediction Server (http://bioinf.cs.ucl.ac.uk/web_servers/psipred_server/disopred_overview/). Structural superpositions were performed with COOT (Secondary Structure Matching option) (Emsley et al., 2010). Amino acid sequence alignments were done using Clustal Omega on the ExPASy server (https://www.ebi.ac.uk/Tools/msa/clustalo/), with corresponding figures prepared with ESPript (http://espript.ibcp.fr/ESPript/ESPript/). Structural representations were done with Chimera (Pettersen et al., 2004).

## 3 Results and discussion

### 3.1 Crystal structure of GG-SpaB

**Fig. 1:**
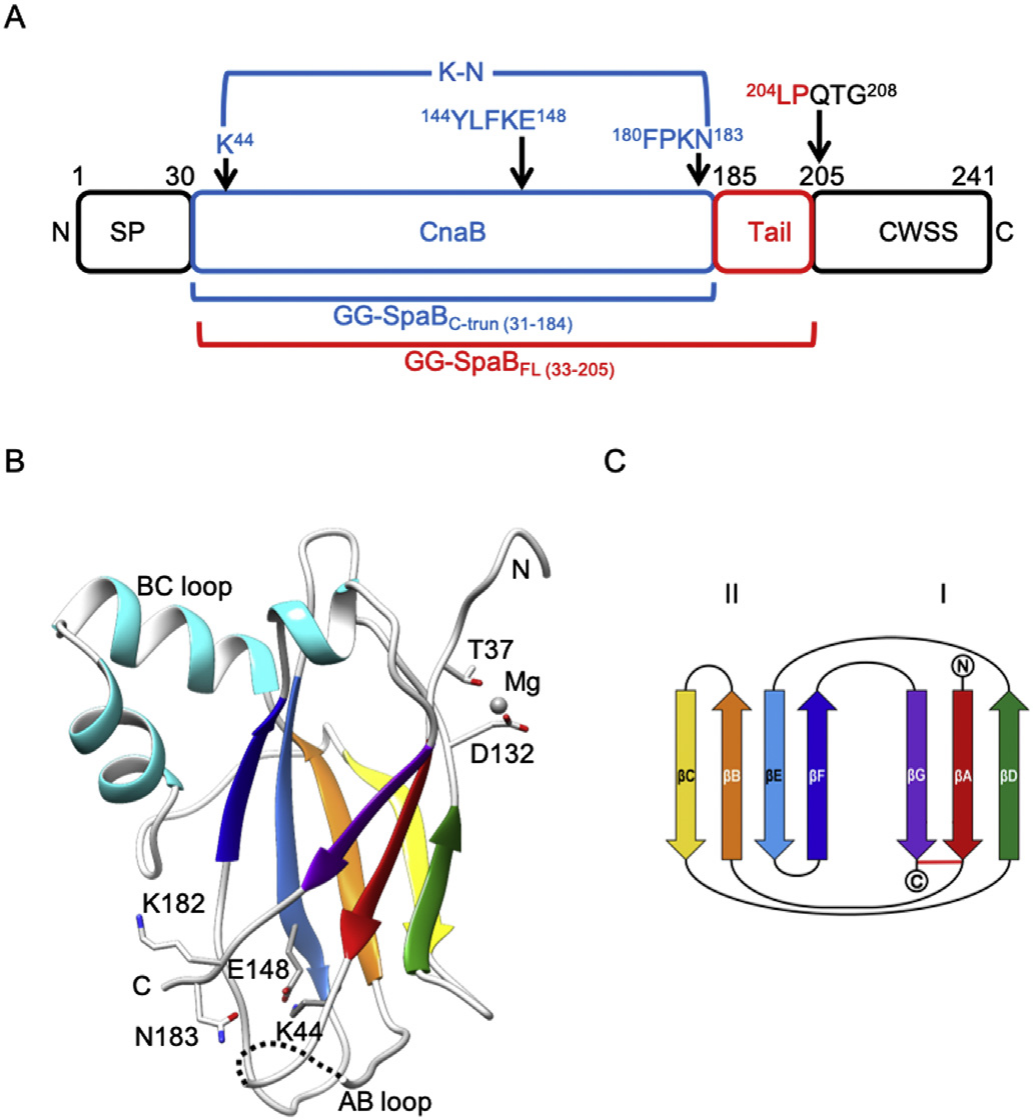
Domain and structural attributes of GG-SpaB. **(A)** Schematic diagram of GG-SpaB depicting its CnaB domain boundaries and associated elements. Locations of the N-terminal signal peptide (SP) and C-terminal cell-wall sorting signal (CWSS) with a pentapeptide motif (LPQTG) are shown. Locations with residue numbers of the potential internal isopeptide bond (K–N) and pilin motif (FPKN) are indicated by upper arrows. Full-length (residues 33–205; GG-SpaB_FL_) and truncated (residues 31–184; GG-SpaB_C-turn_) versions of GG-SpaB are indicated in red and blue, respectively. Locations of the N- and C-termini are marked. **(B)** Ribbon diagram of the GG-SpaB crystal structure. Core β-strands of the CnaB-type fold are indicated using rainbow colors (red to violet). The lengthy BC and AB loops with α-helices (in turquoise) and disorderedness (dotted lines), respectively, are labeled. The autocatalytic triad of residues (K44, N183, and E148) involved in internal isopeptide bond formation are numbered and shown in sticks. The linking lysine (putatively K182) for intermolecular isopeptide bond formation is numbered and shown in sticks. Residues (T37 and D132) involved in coordinating a Mg^2+^ ion are numbered and shown in sticks. Identical residues from the adjacent molecule in the crystal lattice and two water molecules complete the octahedral coordination of Mg^2+^ (see further in Fig. S2). Locations of the N- and C-termini are marked. **(C)** Topology diagram of the β-sandwich fold of the CnaB domain in GG-SpaB. Core β-strands of β-sheets I and II in the CnaB domain are labeled A to G using rainbow colors (red to violet) as in **(B)**. A horizontal red line indicates the approximate position of the residues for the putative K–N isopeptide bond. Locations of the N- and C-termini are marked.

Unprocessed L. *rhamnosus* GG SpaB (GG-SpaB) protein is comprised of 241 amino acids and includes one CnaB domain positioned between a 30-residue N-terminal signal peptide and a 57-residue C-terminal sorting region that contains a LPQTG motif (Fig. 1A). Although a recombinant form of mature full-length GG-SpaB (residues 33–205) was soluble and homogeneous as a C-terminal histidine-tagged protein (GG-SpaB_FL_) (Fig. S1), it did not yield X-ray diffraction quality crystals. Instead, we produced a truncated version of GG-SpaB (GG-SpaB_C-trun_) lacking a flexible part of the C-terminal tail region (residues 185–205) but having a histidine-tag at its N-terminus (Fig. S1). We found the GG-SpaB_C-trun_ protein to be crystallizable following the SLM treatment and the addition of 0.2 ​M MgCl_2_ to the screening condition (Kumar Megta et al., 2019) (Table 1). An X-ray structure was then built from the phases provided by the molecular replacement (MR) method using an assortment of distant structural homologs. Structurally, the GG-SpaB basal subunit has a CnaB domain with approximate dimensions of 42 ​× ​32 ​× ​24 ​Å and a total surface area of 8870 ​Å^2^ (Fig. 1B). The CnaB domain is composed of a core β-sandwich fold that includes three (DAG) and four (CBEF) β-strands (Fig. 1B and C). Prominent loop regions in the structure include the lengthy but disordered AB loop (residues 48–68) between the first and second β-strands, the BC loop as two tandem α-helices, and the FG loop as a short α-helix (Fig. 1B). Interestingly, we observed that one magnesium ion (Mg^2+^), likely derived from the 0.2 ​M MgCl_2_ additive, stabilized the crystal-packing interaction between each pair of molecules in the asymmetric unit of eight monomers of GG-SpaB_C-trun_ (Fig. 1B and Fig. S2). Here, the Thr37 and Asp132 (βA and βD strands, respectively) residues of each molecule, along with two water molecules, are seen to coordinate Mg^2+^ in a typical octahedral geometry (Fig. S2). We suggest that this intermolecular interaction seems to have enhanced the formation of diffraction quality GG-SpaB crystals (Kumar Megta et al., 2019). Finally, as with most other basal and backbone pilins, a pilin-like motif (FPKN) with a solvent-exposed lysine (Lys182) can be found in GG-SpaB. While the pilin-like motif (FPKN) identified in the present study agrees with an earlier-predicted pilin motif (Krishnan et al., 2016), it contradicts another prediction (VSKN) (von Ossowski, 2017), since the putative linking lysine in the latter predicted pilin motif lies on the α-helix of the BC loop and would be unavailable for forming of an intermolecular isopeptide bond (Fig. S3A).

**Table 1:** Data collection and refinement statistics^a^.

Data collection	GG-SpaB_C-trun_
PDB ID	7CBS
X-ray source	BM14, ESRF
Wavelength (Å)	0.97872
Resolution range (Å)	42.40–2.39
Space group	*P*3_2_
Cell dimensions (Å; °)	a ​= ​b ​= ​51.52, c ​= ​408.20; α ​= ​β ​= ​90, γ ​= ​120
No. of unique reflections	41158 (2058)
R_merge_	0.045 (0.428)
R_meas_	0.052 (0.524)
R_*pim*_	0.026 (0.297)
*I*/σ(*I*)	15.0 (2.0)
CC_1/2_	0.99 (0.71)
Completeness (ellipsoidal) (%)	93.2 (72.2)
Multiplicity	3.7 (2.8)
No. of molecules in the asymmetric unit	8
**Refinements**	
*R*_work_/*R*_free_	0.198/0.235
Protein atoms/metal ions/water molecules	7270/4/75
Average B factors (Å^2^)	72
R.m.s deviations - bonds (Å)	0.003
R.m.s deviations - angles (°)	1.27
Ramachandran plot analysis - Residues in favored regions (%)	100

a Values in parentheses are for the outermost resolution shell and where an anisotropic correction was used.

### 3.2 GG-SpaB harbors the residue triad for an internal isopeptide bond

**Fig. 2:**
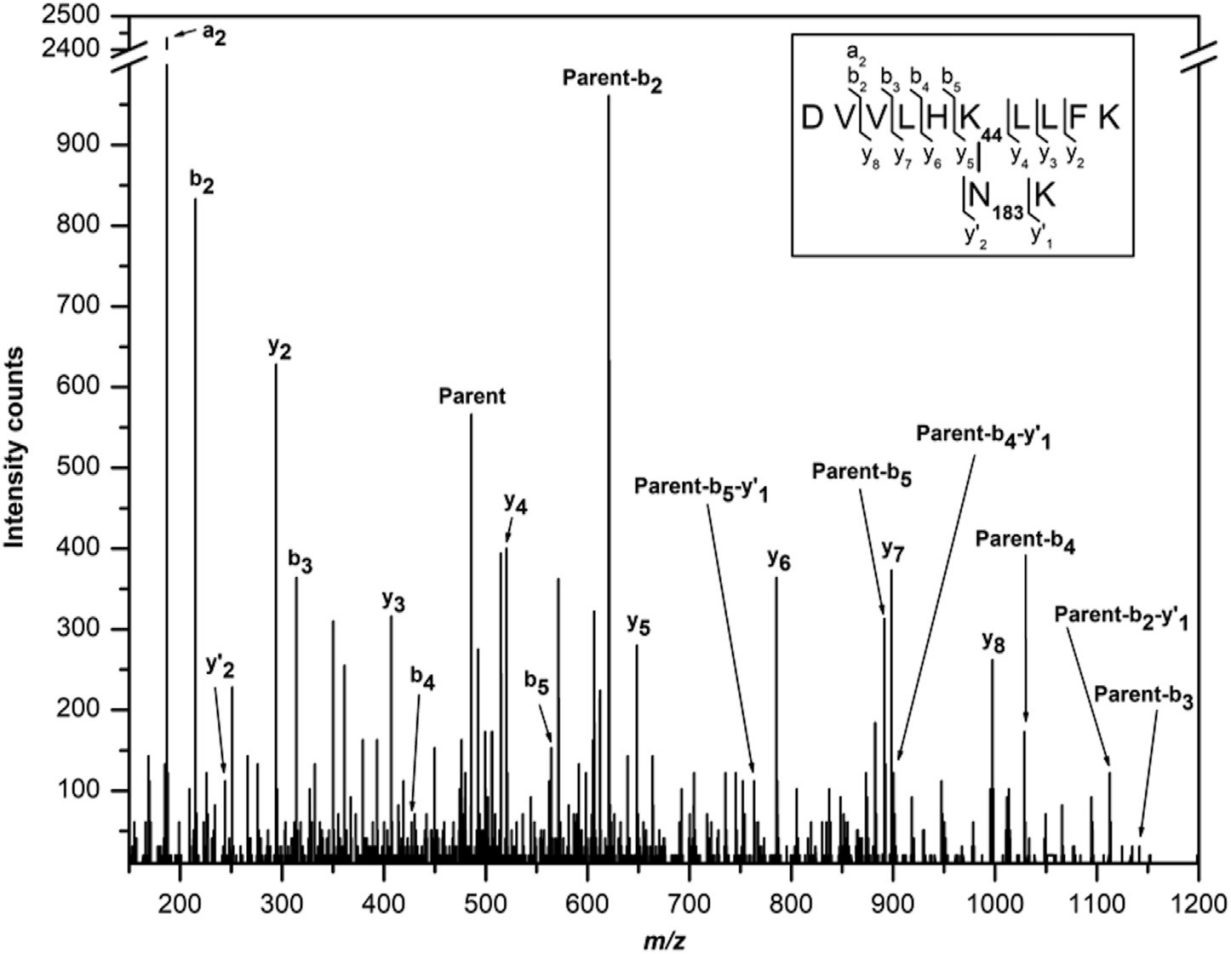
MS/MS spectrum of peptide with mass-to-charge ratio (m/z) 485.62^3+^ generated from trypsin/AspN double digest of GG-SpaB that represent the sequences flanking the internal isopeptide bond formed between lysine (K44) and asparagine (N183) residues. Daughter ions produced are indicated and the proposed structure is shown in the inset.

**Fig. 3:**
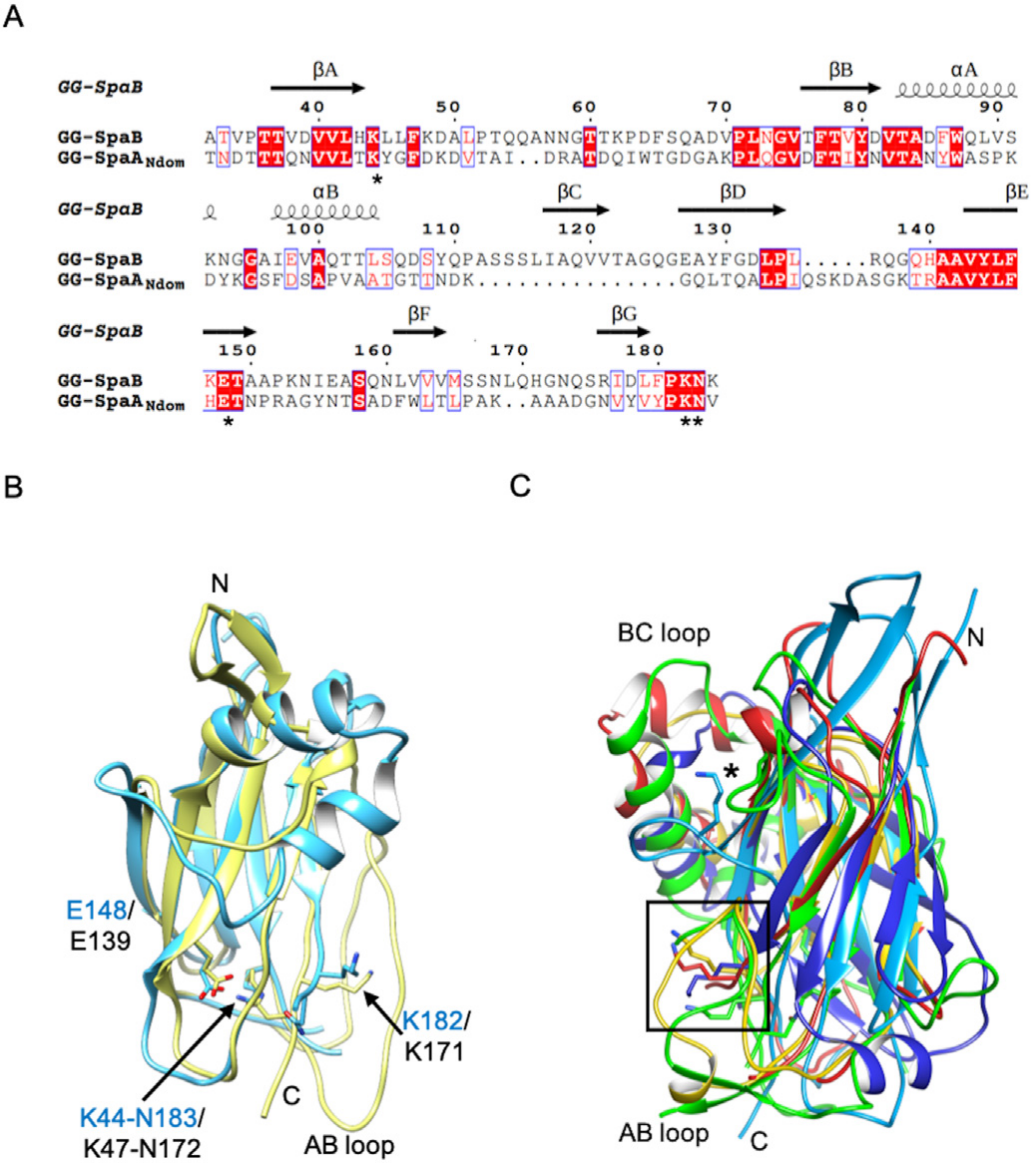
Comparison of GG-SpaB with the N-terminal domain of GG-SpaA (GG-SpaA_Ndom_) and other basal pilins. **(A)** Amino acid sequence alignment between GG-SpaB and GG-SpaA_Ndom_. Core β-strands (A–G) and α-helices are indicated on top. Residues involved with internal and intermolecular isopeptide bond formation are marked by an asterisk (∗). **(B)** Structural superposition of GG-SpaB (cyan) with GG-SpaA_Ndom_ (yellow). The autocatalytic triad of residues for K–N isopeptide bond formation in GG-SpaA_Ndom_ (K47, N172, and E139) and GG-SpaB (K44, N183, and E148) are shown in sticks and labeled. The pilin-motif linking lysines for intermolecular K-T isopeptide bond formation in GG-SpaA_Ndom_ (K171) and GG-SpaB (putatively K182) are shown in sticks and labeled. The lengthy AB loop that covers K171 in GG-SpaA_Ndom_ (hook-shaped) and K182 in GG-SpaB (disordered) is labeled. **(C)** Structural superposition of N-domains from GG-SpaB (red), GG-SpaE (green), GBS52 (gold), RrgC (blue), and FctB (cyan) basal pilins. The location of structurally conserved linking lysines is marked by a square, except for the one at the Ω loop of FctB, which is indicated by an asterisk (∗). The only intact internal isopeptide bond is found in GG-SpaE. Locations of the N- and C-termini are marked.

The crystal structure of GG-SpaB shows that it contains the favored residues for potentially forming an internal K–N isopeptide bond (Fig. 1A and B). Here, the Lys44 and Asn183 residues from the first (A) and last (G) β-strands, respectively, and a proximal autocatalytic Glu148 are located within a hydrophobic core of the CnaB domain. Since these residues adopt a triad configuration, we suggest that isopeptide bond formation should be possible between Lys44 and Asn183. However, as Lys44 and Asn183 are separated by about 5 ​Å, no electron density was available for modeling an intact isopeptide bond that links together these two residues (Fig. S3B). We suspect that increased solvent exposure of the hydrophobic pocket resulting from the C-terminal truncation or then possibly the SLM treatment itself might have prevented the formation of an intact isopeptide bond in the crystal structure of GG-SpaB_C-trun_. On the other hand, our mass spectral analysis of GG-SpaB_FL_ protein samples stored at 4 ​°C for two days revealed the presence of an intact bond (Fig. 2, Fig. S1, and Table S1). Consequently, there is a good possibility that the K–N isopeptide interaction forms more slowly in GG-SpaB_FL_ much like what was observed previously in some other pilins (Kang et al., 2014; Megta et al., 2019). Here, it is also worth mentioning that the key residues needed for forming internal and intermolecular isopeptide bonds are well conserved in both a sequence and structural alignment between GG-SpaB and the closely related backbone GG-SpaA pilin (Fig. 3A and B). The putative linking Lys182 is also structurally conserved in the related basal pilin structures, though these lack an internal isopeptide bond (aside from GG-SpaE) (Fig. 3C).

### 3.3 GG-SpaB resembles the N-terminal domain of the GG-SpaA backbone pilin

To identify the structural homologs of GG-SpaB, we performed a search for similar structures in the protein data bank (PDB) using the DALI server (Holm and Laakso, 2016). Rather expectedly, this revealed that GG-SpaB shares a structural likeness with other basal and backbone pilin subunits. Foremost as the top three hits, these were the N-terminal domains of pilins from *L. rhamnosus* GG, and included GG-SpaA_Ndom_ (PDB ID: 5F44 ​(Chaurasia et al., 2016)), GG-SpaD_Ndom_ (PDB ID: 5YXO ​(Chaurasia et al., 2018)), and GG-SpaE_Ndom_ (PDB ID: 6JCH ​(Megta et al., 2019)). Interestingly, included among these pilins with highest sequence identity was backbone GG-SpaA (Table S2), which, via a K-T intermolecular isopeptide interaction, is connected to GG-SpaB during the assembly of the SpaCBA pilus. In fact, the structural superposition of GG-SpaB and GG-SpaA_Ndom_ gave a rmsd of 1.5 ​Å with 101 residues aligned and 34% sequence identity. Here, we noticed that the spatial relationships among the three residues in the triad configuration for internal K–N isopeptide bond formation in GG-SpaA (Lys47, Asn172, and Glu139) and GG-SpaB (Lys44, Asn183, and Glu148) are structurally conserved (Fig. 3B). Moreover, while the hook-shaped AB loop and the α-helix-containing BC loop appear similar in the GG-SpaB and GG-SpaA structures, there are subtle structural deviations in the DE and FG loops. More conspicuously, we observed that the side chains of the linking lysine in GG-SpaB (putatively Lys182) and the GG-SpaA N-terminal domain (Lys171) (Chaurasia et al., 2016) both point in the same direction at the C-terminal end of each protein (Fig. 3B). Moreover, among the two-metal ion-binding residues in GG-SpaB (Fig. S2), Thr37 is conserved in GG-SpaA (Thr40), though Asp132 lacks a similar counterpart (Chaurasia et al., 2016).

### 3.4 Structural comparison of GG-SpaB with other basal pilins

Previously solved crystal structures of basal pilins (Fig. S4) in the PDB include single-domain FctB (PDB ID: 3KLQ) from *Streptococcus pyogenes* (Linke et al., 2010), two-domain GBS52 (PDB ID: 3PHS) from *Streptococcus agalactiae* (Krishnan et al., 2007) and GG-SpaE (PDB ID: 6JCH) from *L. rhamnosus* GG (Megta et al., 2019), and three-domain RrgC (PDB ID: 4OQ1) from *Streptococcus pneumoniae* (Shaik et al., 2014). However, our DALI search with GG-SpaB did not include FctB and RrgC among the top five hits (Table S2). Alternatively, our manual structural superposition of GG-SpaB with the N-terminal domains of these basal pilins revealed a closer resemblance to GG-SpaE (2.7 ​Å for 170 common Cα atoms with 24% sequence identity) and GBS52 (2.0 ​Å for 95 common Cα atoms with 19% sequence identity) than to FctB (3.5 ​Å for 90 common Cα atoms with 14% sequence identity) and RrgC (3.4 ​Å for 101 common Cα atoms with 9% sequence identity). Here, the AB loop and pilin motif are both well conserved in the GG-SpaB, GG-SpaE, and GBS52 structures. In contrast, a differently oriented AB loop is found in FctB and RrgC. While the position of the linking lysine in GG-SpaB, GG-SpaE, GBS52, and RrgC (lacks a pilin motif) is structurally similar, a differently located lysine is found at the omega (Ω) loop of FctB (Fig. 3C). Interestingly, an intact internal isopeptide is only found in the N-terminal domain of GG-SpaE, though it is a slow-forming one, as also seems the case for GG-SpaB.

### 3.5 Incorporation of GG-SpaB into the SpaCBA pilus

**Fig. 4:**
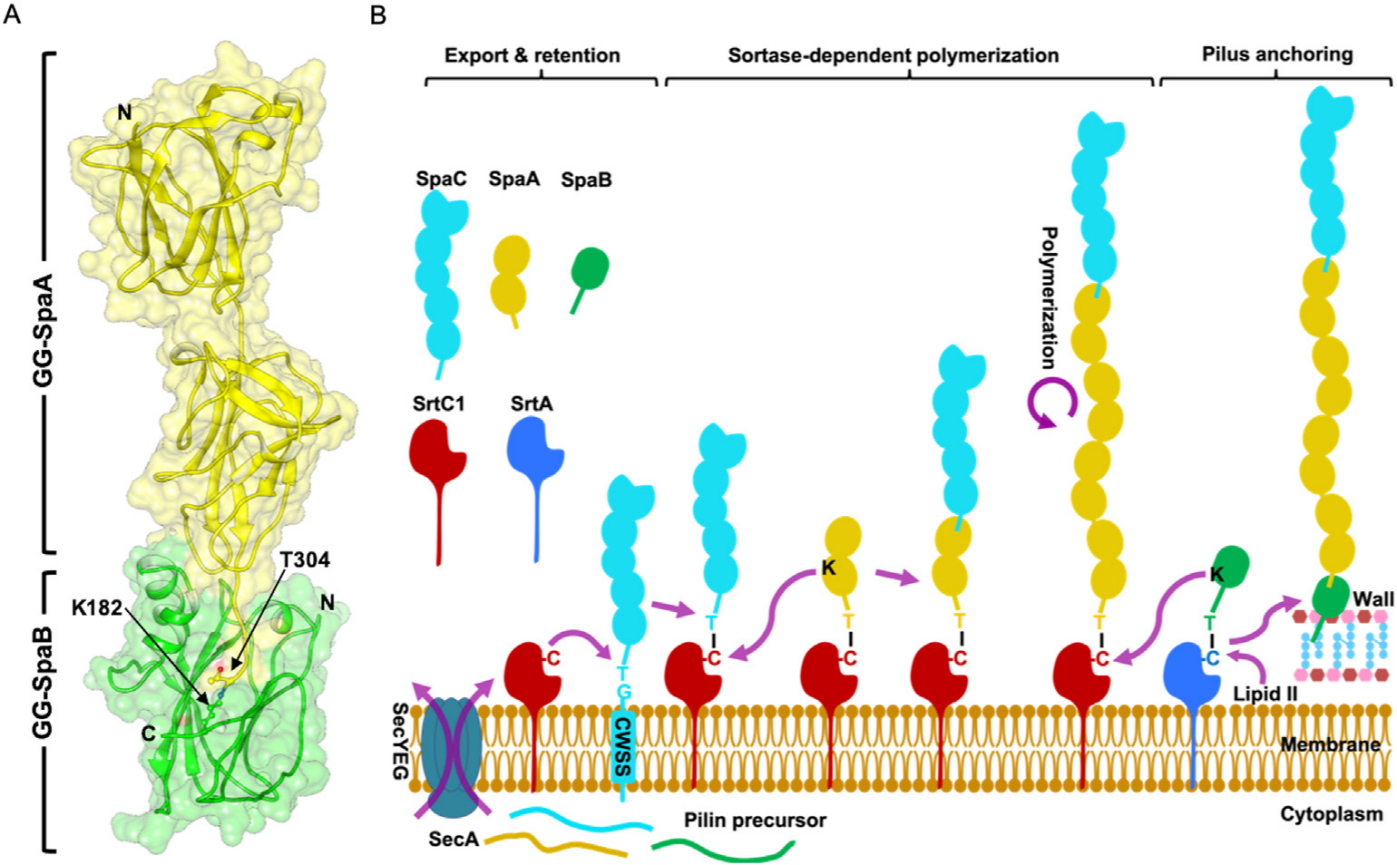
Structural model for incorporating GG-SpaB into the SpaCBA pilus. **(A)** Ribbon and surface representation depicting the interaction between one-domain GG-SpaB and two-domain GG-SpaA. The head-to-tail arrangement of basal GG-SpaB (green) and backbone GG-SpaA (yellow) is based on blind docking between the crystal structures of GG-SpaB (this study) (head) and the C-terminal domain of GG-SpaA (GG-SpaA_Cdom_; PDB ID: 5F44) (tail). Side chains of the threonine (T304) from the LPHTG pentapeptide motif of GG-SpaA (yellow) and the linking lysine (putatively K182) from the FPKN pilin motif of GG-SpaB (green) are within covalent bonding distance and shown in sticks (arrows). Locations of the N- and C-termini are marked. **(B)** Schematic representation of the structural model depicting the incorporation of GG-SpaB during the sortase-mediated assembly of the SpaCBA pilus. SpaCBA pilin precursors with C-terminal sorting signals containing pentapeptide motifs LPHTG or LPQTG are secreted across the cytoplasmic membrane via the Sec system. The C-type sortase SrtC1 (red) cleaves the T858-G859 bond of the LPHTG motif of the preceding tip GG-SpaC subunit (cyan) to produce an acyl-enzyme intermediate (GG-SpaC-SrtC1), which forms by a thioester bond between the sortase active-site cysteine (C223) and T858. Nucleophilic attack from the K171 side chain ε-amino group of the incoming backbone GG-SpaA pilin (yellow) on the GG-SpaC-SrtC1 intermediate yields an intermolecular isopeptide bond (T858-K171) between GG-SpaC and GG-SpaA (Kant et al., 2020). For pilus growth, repetitive nucleophilic attacks from the K171 of the incoming GG-SpaA as a GG-SpaA-SrtC1 intermediate keep adding a succession of GG-SpaA pilins at the base by forming an intermolecular isopeptide bond (T304-K171) between backbone subunits (Chaurasia et al., 2016). Similar to SrtC1, the A-type sortase SrtA (blue) cleaves the T207-G208 bond in the C-terminal LPQTG motif of the basal GG-SpaB subunit (green) to produce an acyl-enzyme intermediate (GG-SpaB-SrtA), which forms by a thioester bond between the sortase active-site cysteine (C201) and T207. Nucleophilic attack from the K182 side chain ε-amino group of the incoming GG-SpaB pilin displaces the GG-SpaA-SrtC1 intermediate, resulting in an intermolecular isopeptide bond (T304-K182) between GG-SpaA and GG-SpaB (present study). With the fully assembled SpaCBA pilus now carried as the acyl-enzyme (GG-SpaB-SrtA) intermediate, it is open to nucleophilic attack from the lipid II precursor and then attached to the cell wall.

As done previously for the basal GG-SpaE pilin (Megta et al., 2019), we performed blind protein-protein docking simulations to provide some structural insight about GG-SpaB incorporation into the SpaCBA pilus. For this, the C-terminal domain of backbone GG-SpaA (GG-SpaA_Cdom_; PDB ID: 5F44) served as the ligand (tail), while basal GG-SpaB served as the receptor (head). Interestingly, the best ranked pose from the docking experiments mimicked the head-to-tail interaction of the symmetry-mates in the crystal packing of the GG-SpaA structure (Chaurasia et al., 2016) (Fig. 4A), in which the long C-terminal tail of GG-SpaA_Cdom_ fits into a hydrophobic groove of GG-SpaB. As we already mentioned before, GG-SpaB resembles the GG-SpaA_Ndom_ structure by having a similarly positioned AB loop, which, together with the core CnaB fold, forms a hydrophobic insertion groove that contains the linking lysine (Fig. 3B). Our previous structural comparisons (see earlier section 3.3, 3.4) had revealed that Lys182 is well positioned for having a putative conjoining role in GG-SpaB, and thus will likely interact with the LPXTG-threonine (Thr304) of GG-SpaA. Notably, though the AB loop in the GG-SpaB_C-trun_ is disordered, this lack of structure is probably due to the absence of the C-terminal tail of GG-SpaA, its natural ligand. Our combination of sequence analysis and molecular dynamic simulation suggests this possibility, as the AB loop appears flexible with a tendency towards disorderedness (Fig. S5). Moreover, since GG-SpaB_C-trun_ had a large portion of its C-terminal tail end truncated away, which was necessary for producing diffraction quality crystals, adjacent molecules in the crystal packing were unable to self-complex with one another, and hence, without this stabilizing influence, the AB loop exists in a disordered state.

Based on a structural analysis (Fig. 4A), we propose a molecular model for SpaCBA pilus assembly in which an intermolecular isopeptide bond forms between Lys182 of GG-SpaB and Thr304 of GG-SpaA and is consistent with GG-SpaB positioning at the pilus base (Fig. 4B). Presumably, the process for incorporating the GG-SpaB subunit at the pilus base is as universally established (Khare and Narayana, 2017). Here, the C-type sortase (SrtC1) cleaves the Thr304-Gly305 bond in the C-terminal LPHTG sorting motif of the preceding GG-SpaA subunit to produce an acyl-enzyme intermediate (GG-SpaA-SrtC1), which is formed by a thioester bond between the sortase active-site cysteine and Thr304. The GG-SpaA-SrtC1 intermediate is likely displaced by nucleophilic attack from the Lys182 side chain ε-amino group of the incoming GG-SpaB, which then results in intermolecular isopeptide bond formation between GG-SpaA and GG-SpaB. It is our contention that the docking process of these two pilins likely follows the three stages of the expose-ligate-seal mechanism, as previously described for the GG-SpaD and GG-SpaE subunits of the SpaFED pilus (Chaurasia et al., 2018; Megta et al., 2019). As part of the process to terminate pilus elongation, GG-SpaB will be carried by an A-type sortase (SrtA) as an acyl-enzyme (GG-SpaB-SrtA) intermediate, and once incorporated at the base of the SpaCBA pilus, it will be susceptible to nucleophilic attack from the lipid II precursor, which would then be followed by the cell-wall attachment of the pilus (Fig. 4B).

Given that earlier immuno-EM results suggested that GG-SpaB was also present along the polymeric backbone of GG-SpaA subunits (Reunanen et al., 2012), we decided to examine the structural evidence for this possibility. Presumably, for GG-SpaB to be structurally sandwiched between two backbone subunits, its C-terminal tail region should resemble that of GG-SpaA. For instance, the GG-SpaA C-terminal region (which includes the residues from the domain boundary to the LPXTG-threonine) that docks with the N-terminal domain of an adjoining GG-SpaA subunit is approximately nine residues in length (^295^DAPSGILPHT^304^). Although our superposition of GG-SpaB with the C-terminal domain of GG-SpaA reveals a similar CnaB fold, wherein a potential internal isopeptide bond occupies a structurally equivalent position, the counterpart C-terminal region in GG-SpaB (^183^NKMVSRHTDAPKKVPKKIRQLLPQT^207^) is 24 residues long and proved to be heavily disordered based on sequence information (Fig. S5 and Fig. S6), which in fact explains the rationale for its removal to promote crystal growth (Kumar Megta et al., 2019). As the C-terminal region in GG-SpaB extends out nearly twice the length as in GG-SpaA, a ten-residue intervening peptide would be introduced between GG-SpaB (tail) and GG-SpaA (head) when these subunits are assembled together. Considering that the GG-SpaB C-terminal region is populated by several positively charged residues, we suggest that any exposure to solvent likely results in the proteolytic degradation of the pilus polymer. In this regard, the presence of GG-SpaB subunits along the pilus backbone would be structurally detrimental and unfavorable, and thus their reported detection by immuno-EM might be an artifactual anomaly, possibly stemming from the cross-reactivity of polyclonal antibodies to GG-SpaB.

### 3.6 C-terminal tail region of GG-SpaB is likely responsible for an atypical mucoadhesiveness

**Fig. 5:**
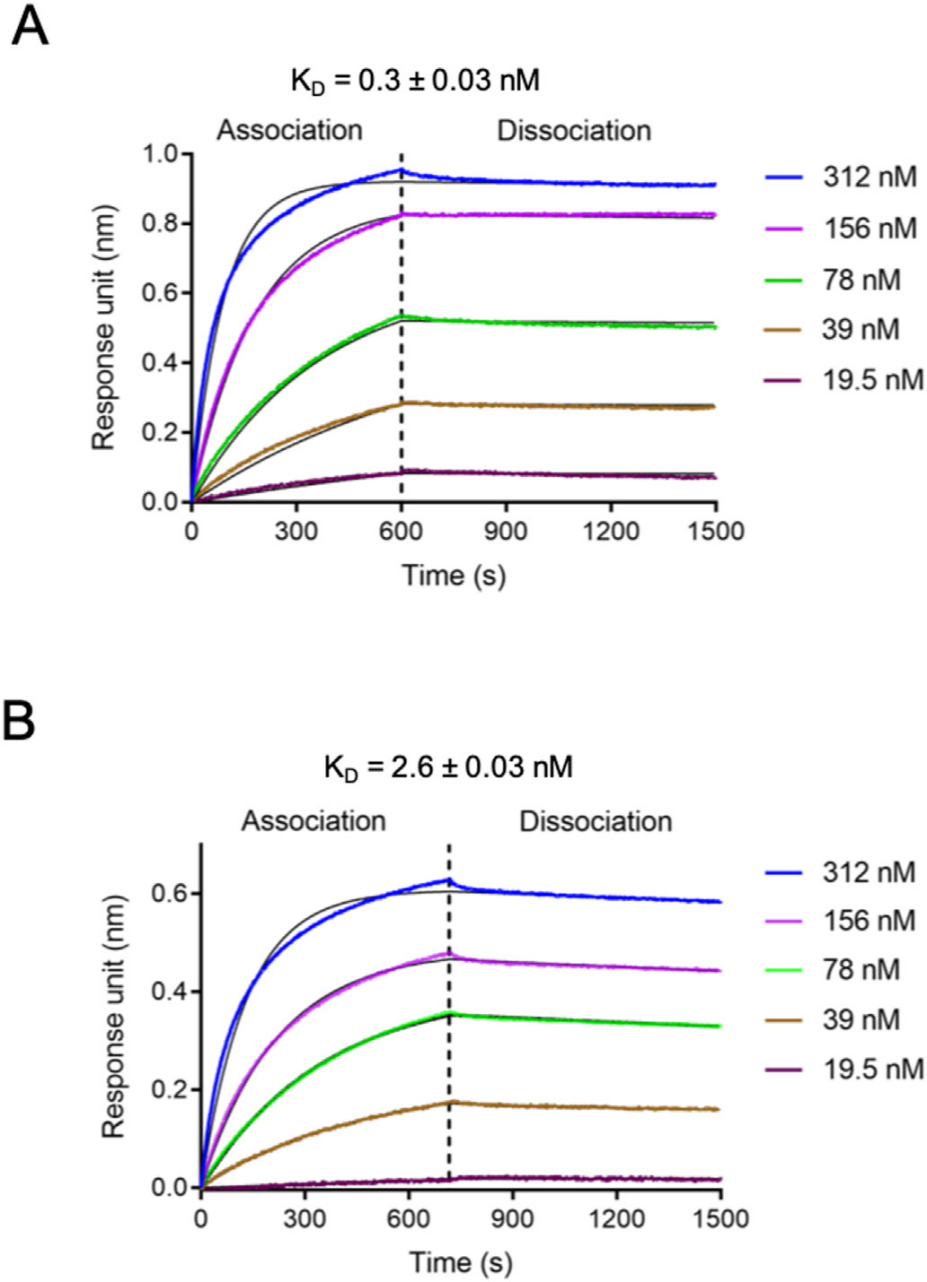
Biolayer interferometry analysis of binding between GG-SpaB proteins and mucin Type II. Biolayer interferometry was carried out with five different concentrations of mucin against immobilized GG-SpaB. **(A)** GG-SpaB_FL_ and mucin Type II. **(B)** GG-SpaB_C-turn_ and mucin Type II.

As mentioned beforehand, the basal GG-SpaB subunit displays a sevenfold greater affinity for intestinal mucus than do the GG-SpaC and GG-SpaF tip adhesins (von Ossowski et al., 2010). Moreover, it has been hypothesized that this mucoadhesive property might be due to electrostatic interactions between positively charged GG-SpaB (pI~8) and negatively charged mucus glycans (von Ossowski et al., 2010). To pinpoint whether the long and positively charged C-terminal region of GG-SpaB plays a role in this atypical binding behavior, we performed biolayer interferometry (BLI) using the GG-SpaB_FL_ (contains the C-terminal region) and GG-SpaB_C-trun_ (lacks the C-terminal region) proteins. Interestingly, among the two proteins, GG-SpaB_FL_ was far more adhesive to mucin Type II than was GG-SpaB_C-trun_ (Fig. 5 and Table S3). Such a result would seem to implicate the C-terminal region as being responsible for the mucoadhesiveness of GG-SpaB.

## 4 Conclusions

In the competitive environs of a microbial microcosm, bacteria often evolve some sort of unique trait that helps them better survive and flourish. One example of such an adaptation is the Gram-positive sortase-dependent pilus, given that this long and adhesive surface appendage is a key factor for the effective colonization of host cells and tissues. Indeed, a large part of the effectiveness lies in the engineered simplicity of its polymeric nature, in which three differently positioned pilin subunits (backbone, tip, and basal) each have a specified function (length, adhesion, and anchoring, respectively) within the pilus structure (Hilleringmann et al., 2009). In ensuing years, X-ray crystallography has had a major role in unraveling and understanding the molecular nuances of these pilin subunits through the solution of their tertiary structures (Krishnan, 2015). With our present study, the crystal structure of the basal GG-SpaB pilin from *L. rhamnosus* GG was determined at 2.39 ​Å resolution. GG-SpaB is much like the other solved structures of basal pilins (GBS52, (Krishnan et al., 2007); FctB, (Linke et al., 2010); GG-SpaE, (Megta et al., 2019); RrgC ​(Shaik et al., 2014),) and assumes a similar CnaB fold, though its own single-domain structure includes a number of distinguishing features. For instance, although GG-SpaB possesses the autocatalytic triad of residues (Lys44, Asn183, and Glu148) necessary for a potential K–N isopeptide interaction, continuous electron density for an intact bond was not visible in the crystal structure. On the other hand, our mass spectral analysis of stored protein suggests that the formation of this internal isopeptide bond occurs likely at a slower pace in GG-SpaB, which then resembles the situation in other pilins such as basal GG-SpaE (Megta et al., 2019) or *Corynebacterium diphtheriae* backbone SpaD (Kang et al., 2014). Still, if the K–N isopeptide bond were to actually form in the context of a fully assembled pilus, this covalent interaction would increase the structural rigidity of GG-SpaB, thereby providing some protection from the proteolytic activity of the gut where SpaCBA-piliated *L. rhamnosus* GG cells inhabit.

Interestingly, we found that GG-SpaB harbors the necessary elements, i.e., pilin-motif linking lysine (putatively Lys182) and flexible AB loop, for a covalent intermolecular isopeptide linkage to the C-terminal tail of the backbone GG-SpaA pilin (and thus the expected incorporation at the base of the SpaCBA pilus). Here, our structural evidence suggests that the docking of GG-SpaB (head) to GG-SpaA (tail) at the pilus base is likely to follow the three-stage process of the expose-ligate-seal mechanism, as we proposed previously for GG-SpaD and GG-SpaE of the SpaFED pilus (Chaurasia et al., 2018; Megta et al., 2019). On the other hand, the aberrant properties of the GG-SpaB C-terminal region seem to not favor the reverse assembly of the GG-SpaB (tail) and GG-SpaA (head) pilins. Because the C-terminal region of GG-SpaB is much longer and more positively charged than that of GG-SpaA, a ten-residue intervening peptide susceptible to proteolytic attack would exist between these two subunits, thus making the pilus too structurally unstable and fragile. Given this possibility, we regard the previous immuno-EM result suggesting that GG-SpaB sandwiches itself between two GG-SpaA subunits in the SpaCBA pilus (Reunanen et al., 2012) might be an artifactual observation due to the cross-reactivity of the polyclonal antibodies being used.

Of further interest, since the C-terminal tail of GG-SpaB is well-laden with positively charged lysine and arginine residues, we consider that this marks a clear contrast with the same region of many other basal pilins, which instead is enriched with hydrophobic prolines and thought to have some structural role in the cell-wall anchoring of the pilus (Linke et al., 2010). Nonetheless, in our opinion a certainly remarkable finding was that the atypical mucoadhesiveness of GG-SpaB (von Ossowski et al., 2010) stems from the positive charged character of its C-terminal region, in which electrostatic contacts are presumably responsible for the binding with negatively charged mucus glycans. However, the actual biological relevance of mucoadhesive GG-SpaB still remains unresolved, as this same binding ability appears to go undetected in the fully assembled SpaCBA pilus (von Ossowski et al., 2010; von Ossowski et al., 2013).

Finally, with the tertiary structure determination of the GG-SpaC (Kant et al., 2020), GG-SpaB (this study), and GG-SpaA (Chaurasia et al., 2016) pilins now in place, our future work will involve using cryo-electron microscopy to reconstruct the overall macromolecular architecture of the native SpaCBA pilus structure.

## CRediT authorship contribution statement

**Abhin Kumar Megta:** performed the cloning, purification, crystallization, mass spectrometry experiments, solved the crystal structure and performed final refinements, performed BLI experiments, wrote the initial draft. **Shivendra Pratap:** solved the crystal structure and performed final refinements, performed BLI experiments, wrote the initial draft. **Abhiruchi Kant:** performed BLI experiments. **Airi Palva:** constructed and provided the expression clone for recombinant GG-SpaBFL.. **Ingemar von Ossowski:** constructed and provided the expression clone for recombinant GG-SpaBFL, revised and finalized the manuscript for submission. **Vengadesan Krishnan:** planned and supervised the project, collected crystal data, wrote the initial draft, revised and finalized the manuscript for submission.

## Declaration of competing interest

The authors declare that they have no known competing financial interests or personal relationships that could have appeared to influence the work reported in this paper.
