# Supplementary material for: Crystal structure of the atypically adhesive SpaB basal pilus subunit: Mechanistic insights about its incorporation in lactobacillar SpaCBA pili
Source: Curr Res Struct Biol. 2020 Dec 8;2:229–38. doi: 10.1016/j.crstbi.2020.11.001 (PMC8244301; doi:10.1016/j.crstbi.2020.11.001)
Supplement: Multimedia component 1 [file mmc1.pdf]

Supplementary data:

Crystal structure of the atypically adhesive SpaB basal pilus subunit: Mechanistic insights about its incorporation in lactobacillar SpaCBA pili

Abhin Kumar Megta [a,b], Shivendra Pratap [a], Abhiruchi Kant [a,c], Airi Palva [d], Ingemar von Ossowski [d,1], and Vengadesan Krishnan [a,*]

[a] Laboratory of Structural Microbiology, Regional Centre for Biotechnology, NCR Biotech Science Cluster, Faridabad 121001, India
[b] School of Biotechnology, KIIT University, Odisha 751024, India
[c] Department of Biotechnology, Manipal University, Karnataka 576104, India
[d] Department of Veterinary Biosciences, University of Helsinki, Helsinki FIN-00014, Finland

[*] **Corresponding author.** Laboratory of Structural Microbiology, Regional Centre for Biotechnology, NCR Biotech Science Cluster, P.O. Box No. 3, Faridabad 121001, India, Tel. +911292848825.
Email address: kvengadesan@rcb.res.in (V. Krishnan).

[1] **Present address.** Department of Bioproducts and Biosystems, School of Chemical Engineering, Aalto University, 02150 Espoo, Finland.

A

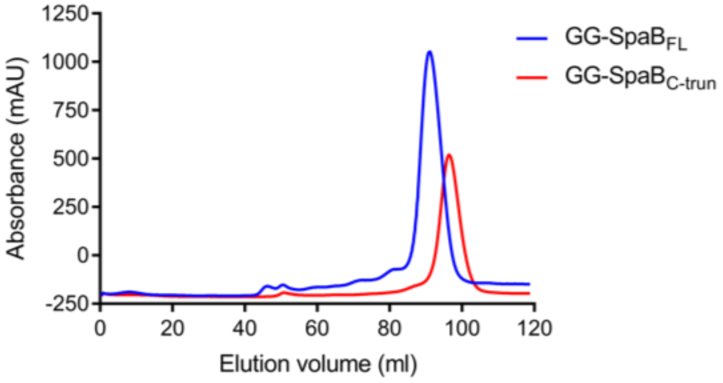

B

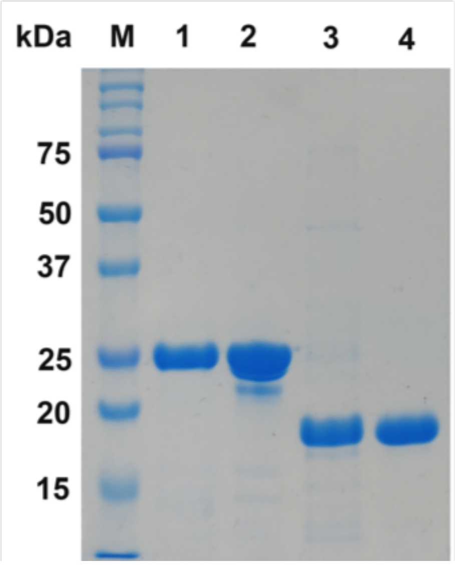

Fig. S1. Size-exclusion chromatography and SDS-PAGE analysis of GG-SpaB proteins. **(A)** Size exclusion chromatographic elution profile of GG-SpaB$_{FL}$ and GG-SpaB$_{C\text{-}trun}$ proteins on HiLoad 16/600 Superdex 200 pg (GE Healthcare) with an elution volume of 91.26 ml and 96.76 ml, respectively. **(B)** SDS-PAGE (15%) analysis of GG-SpaB after purification by size-exclusion chromatography. Freshly purified GG-SpaB$_{FL}$ (Lane 1). GG-SpaB$_{FL}$ stored at 4°C for two days (Lane 2). Freshly purified GG-SpaB$_{C\text{-}trun}$ (Lane 3). GG-SpaB$_{C\text{-}trun}$ stored at 4°C for two days (Lane 4). Molecular weight of markers (M) is shown on the left side of the gel.

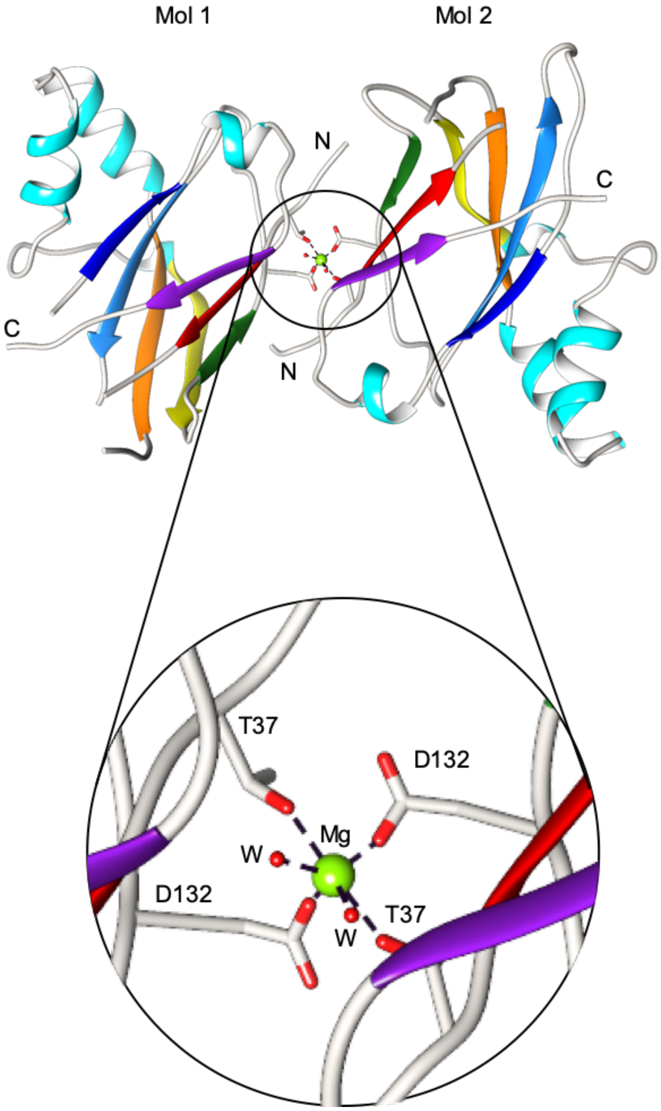

Fig. S2. Magnesium ion stabilization of the intermolecular interaction between the two molecules in the crystal lattice of GG-SpaB$_{C\text{-trun}}$. Eight molecules in the asymmetric unit are arranged into four pairs, with each pair stabilized by a magnesium ion. Ribbon diagram of a pair of GG-SpaB$_{C\text{-trun}}$ molecules (Mol 1 and Mol 2, respectively) are shown in rainbow colors (red to violet). Locations of the N- and C-termini are marked. An enlarged view depicts Mg^{2+} ion coordination in a typical octahedral geometry that includes two key residues (T37 and D132) from each GG-SpaB$_{C\text{-trun}}$ molecule (in sticks and numbered) and two water molecules.

A

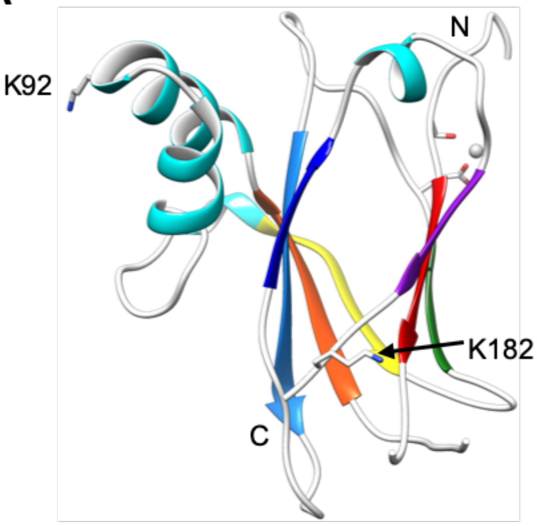

B

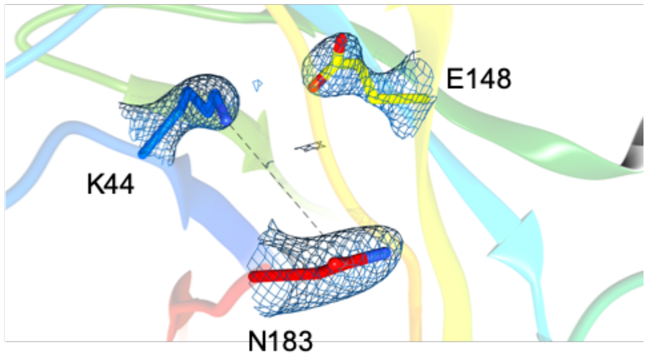

Fig. S3. Structural location of lysine residues involved with intermolecular and internal isopeptide bond formation in GG-SpaB. **(A)** Location of the putative intermolecular isopeptide bond-linking lysine. K182 from the predicted FPKN pilin motif (this study and Krishnan et al., 2016) is located at the C-terminal end adjacent to the flexible (disordered) AB loop. K92 from another predicted pilin motif (VSKN) (von Ossowski, 2017) lies on the α-helix in the BC loop. **(B)** The electron density (2Fo-Fc) map (contoured at 1.5σ) around the conserved residues for the formation of the internal K-N isopeptide bond in GG-SpaB. The autocatalytic triad of residues for internal isopeptide bond formation (K44, N183, and E148) are shown in sticks and numbered. The distance (dashed line) between the N atom of the K44 side chain ε-amino group and the Cγ atom of the N183 side chain is about 5 Å. This distance is similar for all eight molecules in the asymmetric unit.

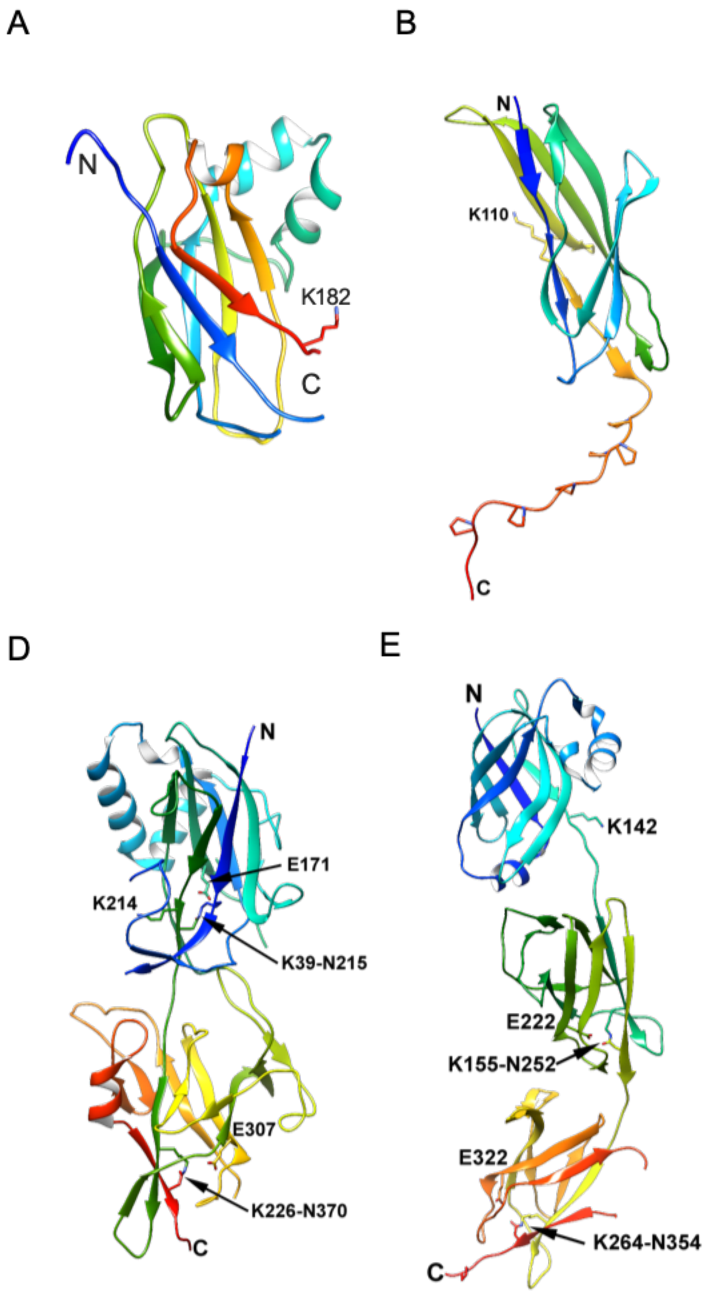

Fig. S4. Structural comparison between GG-SpaB and related basal pilins. Ribbon diagram of basal pilin crystal structures are shown in blend through colors, starting with blue at the N-terminal (N) and ending with red at the C-terminal (C). Linking lysines, isopeptide bond triad residues, and C-terminal prolines are shown in sticks. **(A)** Single-domain GG-SpaB from *L. rhamnosus* GG (this study). **(B)** Single-domain FctB from *S. pyogenes* (PDB ID: 3KLQ). **(C)** Two-domain GBS52 from *S. agalactiae* (PDB ID: 3PHS). **(D)** Two-domain GG-SpaE from *L. rhamnosus* GG (PDB ID: 6JCH). **(E)** Three-domain RrgC from S. *pneumoniae* (PDB ID: 4OQ1). Locations of the loops and the N- and C-termini are marked.

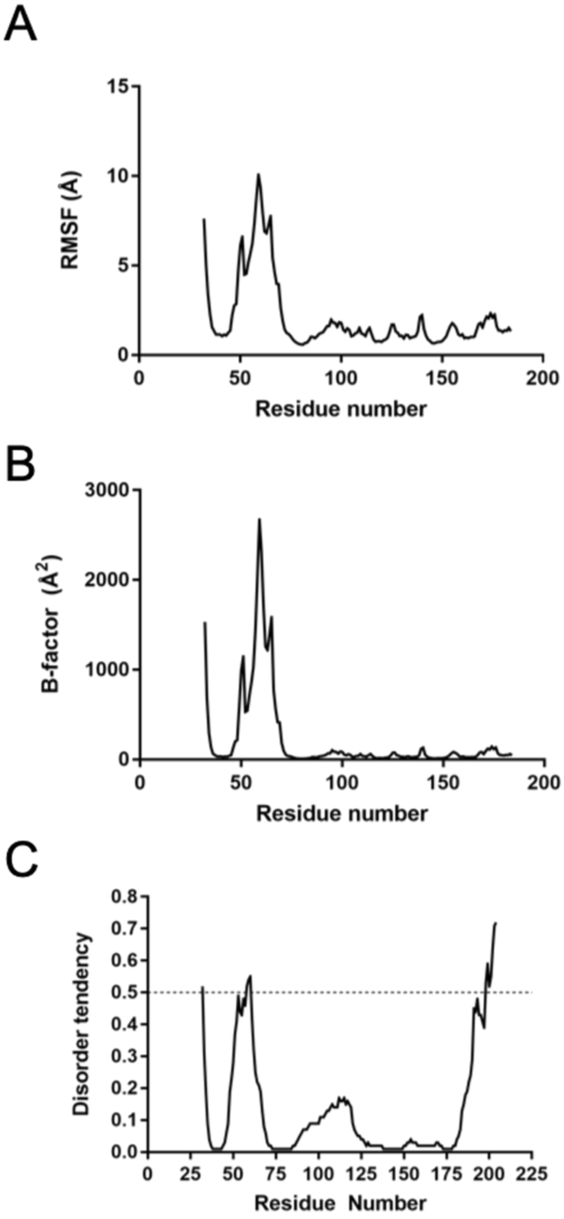

Fig. S5. Flexibility analysis of the AB loop in GG-SpaB. **(A)** RMSF of residues in GG-SpaB during MD simulations. Residues with maximum RMSF values are within the AB loop (peak). **(B)** B-factors of residues in GG-SpaB during MD simulations. Residues with maximum B-factor values are within the AB loop (peak). **(C)** Disorder prediction profile of GG-SpaB. Regions corresponding to the AB loop with protein disorder above the 0.5 confidence level (dotted horizontal line) are indicated.

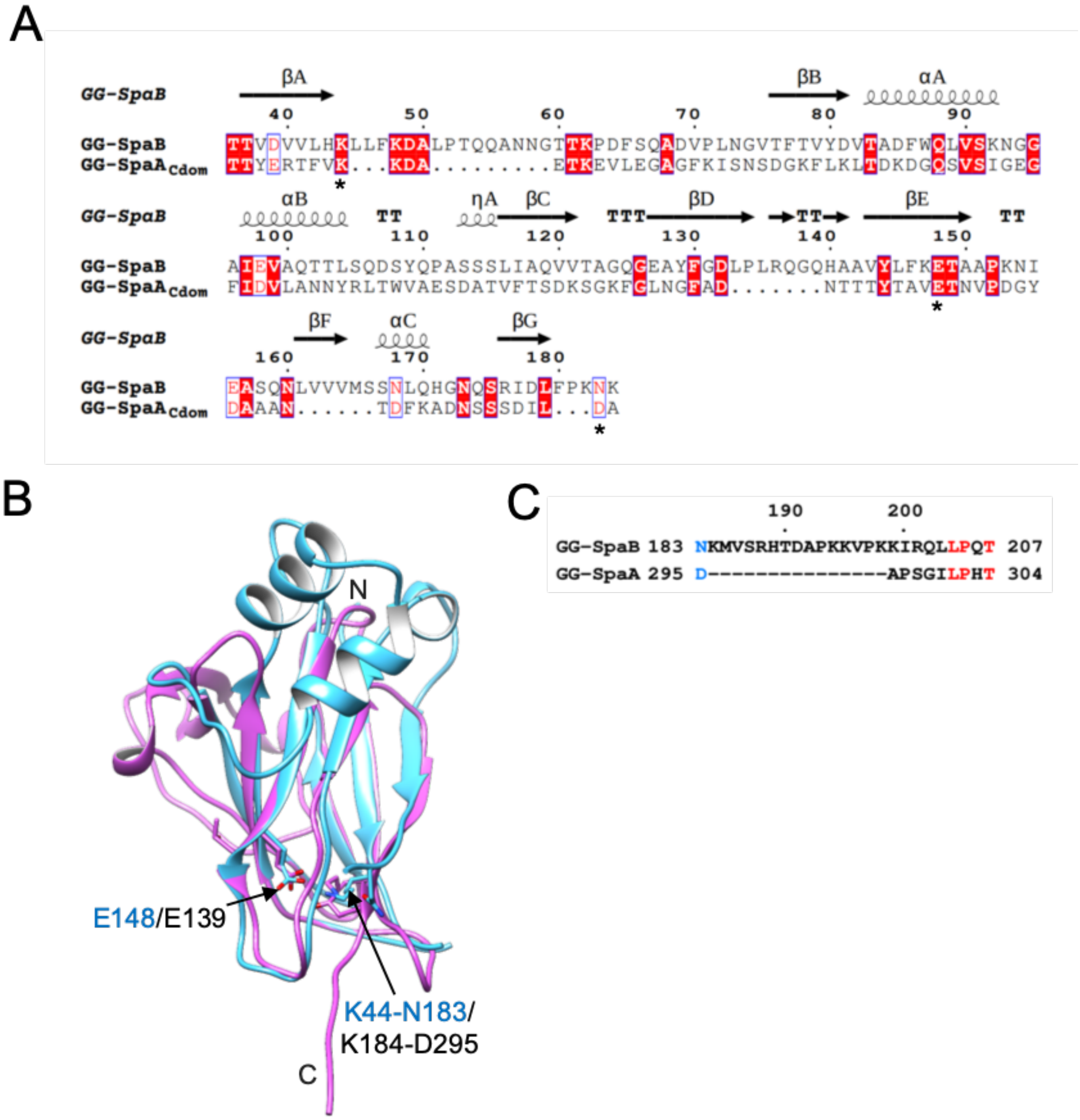

Fig. S6. Comparison of GG-SpaB with the GG-SpaA C-terminal domain (GG-SpaA$_{Cdom}$). **(A)** Amino acid sequence alignment between GG-SpaB and GG-SpaA$_{Cdom}$. Core β-strands (A-G) and α-helices are indicated on top. Residues involved in isopeptide bond formation are marked by an asterisk (*). **(B)** Structural superposition of GG-SpaB (cyan) with GG-SpaA$_{Cdom}$ (violet). The catalytic triad of residues for internal isopeptide bond formation in GG-SpaA$_{Cdom}$ (K184, D295, and E269) and GG-SpaB (K44, N183, and E148) are shown in sticks and numbered. Locations of the N- and C-termini are marked. **(C)** Amino acid sequence alignment of the C-terminal tail regions of GG-SpaB and GG-SpaA$_{Cdom}$. Isopeptide bond residues N183 (GG-SpaB) and D295 (GG-SpaA$_{Cdom}$) are in light blue. LPXTG-like pentapeptide motifs for GG-SpaB and GG-SpaA$_{Cdom}$ are shown in red.

Table S1. MS/MS analysis of crosslinked peptide at *m/z* 485.62^{3+} containing an internal isopeptide bond between lysine (K44) and asparagine (N183) residues in proximity of an autocatalytic glutamate (E148). Daughter ions of the fragmentation of the parent ion (DVVLHK$_{44}$LLFK and N$_{183}$K) are listed.

Observed (m/z) [a]	Charge	Calculated (m/z) [b]	$\Delta_{obs-calc}$	Proposed structure	Ion type
187.10	+1	187.10	0	DV	a_2
215.10	+1	215.10	0	DV	b_2
244.12	+1	244.12	0	NK (-NH_3)	y'_2
294.18	+1	294.18	0	FK	y_2
314.17	+1	314.17	0	DVV	b_3
407.26	+1	407.26	0	LFK	y_3
427.25	+1	427.25	0	DVVL	b_4
485.62	+3	485.63	-0.01	DVVLHKLLFK and NK (-NH_3 [c]) [d]	Parent
520.34	+1	520.34	0	LLFK	y_4
564.31	+1	564.31	0	DVVLH	b_5
620.89	+2	620.89	0	VLHKLLFK and NK (-NH_3 [c])	Parent-b_2
648.44	+1	648.44	0	KLLFK	y_5
763.46	+1	763.47	-0.01	KLLFK and N (-NH_3 [c])	Parent-b_5-y'_1
785.50	+1	785.50	0	HKLLFK	y_6
891.56	+1	891.58	-0.02	KLLFK and NK (-NH_3 [c])	Parent-b_5
898.58	+1	898.58	0	LHKLLFK	y_7
900.53	+1	900.53	0	HKLLFK and N (-NH_3 [c])	Parent-b_4-y'_1
997.65	+1	997.65	0	VLHKLLFK	y_8
1028.62	+1	1028.62	0	HKLLF and NK (-NH_3 [c])	Parent-b_4
1112.68	+1	1112.69	-0.01	VLHKLLFK and N (-NH_3 [c])	Parent-b_2-y'_1
1141.70	+1	1141.72	-0.02	LHKLLFK and NK (-NH_3 [c])	Parent-b_3

[a] Monoisotopic mass of observed ions.
[b] Monoisotopic masses were calculated using the Fragment Ion Calculator (https://db.systemsbiology.net:8080/proteomicsToolkit/FragIonServlet.html).
[c] Loss of 17 Da due to asparagine deamination is shown in parenthesis.
[d] Unfragmented parent ion.

Table S2. Top five structural homologs identified by a DALI search of the PDB.

Sl. No.	Chain	Z-score	RMSD	Alignment length (aa)	No. of residues	Sequence identity (%)	PDB ID	Description
1.	A	17.1	1.6	117	415	30	5YXO	*L. rhamnosus* GG SpaD backbone pilin
2.	C	12.3	2.1	105	256	34	5F44	*L. rhamnosus* GG SpaA backbone pilin
3.	A	11.1	2.7	115	327	24	6JCH	*L. rhamnosus* GG SpaE basal pilin
4.	A	10.6	2.7	107	449	21	3UXF	*Actinomyces oris* FimP backbone pilin
5.	A	10.4	2.1	95	238	18	3PHS	*Streptococcus agalactiae* GBS52 basal pilin

Table S3. Estimation of binding affinity between GG-SpaB proteins (ligand) and mucin type II (analyte) via biolayer interferometry. Equilibrium dissociation constants (K_D) for the ligand/analyte complexes are determined. Binding assays are performed in duplicate. Standard error of mean values (±) are included.

Ligand	Analyte	K_D (M) 1:1 binding	K_{on} (1/Ms)	K_{dis} (1/S)	Full χ^2	Full R^2
GG-SpaB_{FL}	Mucin type II	$3.06 \times 10^{-10} \pm 0.03$	$3.53 \times 10^{4} \pm 0.02$	$1.09 \times 10^{-5} \pm 0.01$	1.31	0.9983
GG-Spa$B_{C\text{-}trun}$	Mucin type II	$2.55 \times 10^{-9} \pm 0.03$	$2.63 \times 10^{4} \pm 0.02$	$6.86 \times 10^{-5} \pm 0.03$	0.71	0.9963
